# Effect of saturated fatty acid-rich dietary vegetable oils on lipid profile, antioxidant enzymes and glucose tolerance in diabetic rats

**DOI:** 10.4103/0253-7613.66835

**Published:** 2010-06

**Authors:** Benson Mathai Kochikuzhyil, Kshama Devi, Santosh Raghunandan Fattepur

**Affiliations:** Department of Pharmacology, Al-Ameen College of Pharmacy, Bangalore, India

**Keywords:** Antioxidant enzymes, atherogenicity, coconut oil, glucose tolerance, palm oil, saturated fatty acid

## Abstract

**Objective:**

To study the effect of saturated fatty acid (SFA)-rich dietary vegetable oils on the lipid profile, endogenous antioxidant enzymes and glucose tolerance in type 2 diabetic rats.

**Materials and Methods:**

Type 2 diabetes was induced by administering streptozotocin (90 mg/kg, i.p.) in neonatal rats. Twenty-eight-day-old normal (N) and diabetic (D) male Wistar rats were fed for 45 days with a fat-enriched special diet (10%) prepared with coconut oil (CO) – lauric acid-rich SFA, palm oil (PO) – palmitic acid-rich SFA and groundnut oil (GNO) – control (N and D). Lipid profile, endogenous antioxidant enzymes and oral glucose tolerance tests were monitored.

**Results:**

D rats fed with CO (D + CO) exhibited a significant decrease in the total cholesterol and non-high-density lipoprotein cholesterol. Besides, they also showed a trend toward improving antioxidant enzymes and glucose tolerance as compared to the D + GNO group, whereas D + PO treatment aggravated the dyslipidemic condition while causing a significant decrease in the superoxide dismutase levels when compared to N rats fed with GNO (N + GNO). D + PO treatment also impaired the glucose tolerance when compared to N + GNO and D + GNO.

**Conclusion:**

The type of FA in the dietary oil determines its deleterious or beneficial effects. Lauric acid present in CO may protect against diabetes-induced dyslipidemia.

## Introduction

Dietary fatty acids (FAs) play a key role in various pathological processes involved in diabetes mellitus (DM), such as insulin resistance and atherothrombogenic risk.[1–5] Both DM and dietary fats have a significant role in the development of dyslipidemia and atherosclerosis by modulating the serum lipid profile[6] and free radical generation.[7] Consumption of excess and wrong dietary saturated fats under DM conditions can accelerate the atherosclerotic process and is considered to be harmful.[1–3] Therefore, consumption of saturated fatty acid (SFA)-rich dietary vegetable oils, viz. coconut oil (CO) and palm oil (PO) is discouraged.[3,8] Medicinal uses of CO are mentioned in ayurvedic texts, which characterize CO as “hrdyam” or “good for heart and cardiovascular system.”[3,9] Whereas PO, another SFA-rich oil, is widely used in developing countries, including India, due to its low cost.[3] There are reports both in favor and in opposition to the health effects of CO and PO on coronary heart disease and DM.[3] Hence, the present study was carried out to investigate the influence of CO and PO on the lipid profile, endogenous antioxidant enzymes and glucose tolerance in experimentally induced type 2 DM rats.

## Materials and Methods

### Experimental animals

In-house laboratory bred male Wistar rats of 4 weeks of age (45 ± 3 g) were selected for the study. The animals were housed in polypropylene cages on clean paddy husk beddings and were maintained under controlled temperature of 20° ± 2°C with an alternating 12-h light:dark cycle (light on 6.00-18.00 h). Diet and water were provided ad libitum. The experimental protocol was approved by the Institutional Animal Ethics Committee (IAEC). Animal ethical guidelines and good laboratory practice guidelines were followed throughout the experimental period. In addition, all the precautions were taken to minimize pain and discomfort to the animals.

### Test dietary fats

Groundnut oil (GNO), CO and PO were purchased from the local market.

### Test diets

Diet 1: GNO (Control); Diet 2: CO (lauric acid-rich SFA); Diet 3: PO (palmitic acid-rich SFA). The diets were prepared according to the modified American Institute of Nutrition formulae (AIN-76).[2,3] GNO was chosen for the control diets because it is the most commonly used edible oil in Southern India. The dose of the dietary vegetable oil was 10% (w/w). Diets were stored in a refrigerator (2°–8°C) and were prepared freshly every week. Individual groups (n = 6) were fed with the respective diet for 45 days. Animals were provided with fresh diet daily ad libitum and the left over food was discarded.

### Experimental conditions

### Normal (N) group

Rats were maintained under standard laboratory conditions and fed with the respective diets till the completion of the experiment.

### Diabetes (D) group

A mild and stable form of diabetes resembling type 2 human DM was produced by intraperitoneal injection of a freshly prepared solution of Streptozotocin (90 mg/kg) to 2-day-old rat pups.[10] On the 27^th^ day, the rats were subjected to an oral glucose tolerance test (OGTT). At the end of 1 h of glucose challenge, the rats showing glucose levels 185 ± 15 mg/dl (against sham control glucose level <100 mg/dl) were included in the study. Later, 28-day-old rats were fed with the respective diets till the completion of the experiment.

### Biochemical analysis

At the end of 45 days, 2.0 ml of blood was withdrawn from the orbital sinus and the serum was separated by centrifuging at 6000 rpm for 15 min. Lipid profile, viz. total cholesterol (TC), high-density lipoprotein cholesterol (HDL-C) and triglyceride (TG) were estimated by biochemical kits from Ranbaxy, India using a semiautoanalyser, artos from Switzerland. Non-HDL-C and atherogenic index (AI) were calculated according to the following formulae:

 Non-HDL-C = TC-HDL-C[2]

 AI = TC-HDL-C/HDL-C[2]

### OGTT,

A dose of 1 g/kg of glucose solution (50%) was administered orally to animals that were fasted overnight.[11] Blood was withdrawn at 0, 15, 30, 60, 120 and 240 min and the glucose levels were determined using a biochemical kit (Enzokit; Ranbaxy, India) on the semiautoanalyser.

### Activity of endogenous antioxidant enzymes

Animals were sacrificed by cervical dislocation. Livers of the animals were perfused with normal saline and were dissected and processed. Ten percent homogenates were prepared in saline (10%w/v) and centrifuged and the supernatant was used for antioxidant enzyme assays. The total protein content, lipid peroxidation (LPO), catalase and superoxide dismutase (SOD) activity were determined using standard procedures.[2,3]

### Statistical analysis

The results were expressed as mean ± SEM (n = 6). The statistical analysis involving four groups was performed by one-way analysis of variance (ANOVA) followed by Dunnett’s test. *P*-value at < 0.05 was considered as statistically significant. All the data were processed with Graph Pad Prism version 5.00 software.

## Results

### Effect on the serum lipid profile

**Figure 1:**
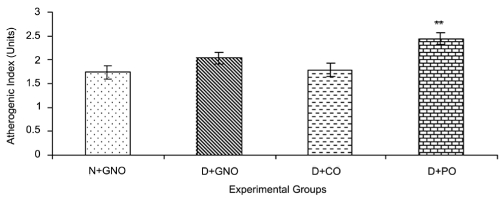
Effect of saturated fatty acid-rich dietary vegetable oils on the atherogenic index in diabetic rats. Bars represent mean values ± SEM of six rats in each group; **P < 0.01 as compared to the N + GNO diet (by one-way ANOVA followed by Dunnett’s multiple comparison test); N + GNO = normal rats fed with groundnut oil; D + GNO = diabetic rats fed with groundnut oil; D + CO = diabetic rats fed with coconut oil; D + PO = diabetic rats fed with palm oil

The D + GNO (D control) group showed a significant increase in the lipid profile, namely TC (*P* < 0.001), HDL-C (*P* < 0.05) and non-HDL-C (*P* < 0.01) as compared with the N + GNO (N control) group. However, the D control group had an apparent increase in AI. Supplementation of CO and PO to D rats showed differential effects. While the CO treatment showed an apparent reduction in AI as compared to the N control, the PO treatment significantly enhanced (*P* < 0.001) the TC, TG and AI as compared to the N control. Moreover, CO administration significantly reversed the D-associated dyslipidemia with respect to the TC (*P* < 0.01) and non-HDL-C (*P* < 0.05) levels whereas PO administration increased the D-associated hyperlipemia (*P* < 0.01) [Table 1 and Figure 1].

**Table 1:** Effect of saturated fatty acidrich dietary vegetable oils on the lipid profile in normal and diabetic rats (n = 6)

*Parameters*	*N* + *GNO*	*D* + *GNO*	*D* + *CO*	*D* + *PO*
TC*f*	61.86 ± 1.58	77.99 ± 2.17***	67.17 ± 2.14##	81.34 ± 2.49***
TG*f*	118.3 ± 2.54	131.5 ± 4.59	121.6 ± 3.0	152.7 ± 4.41***,##
HDL-C*f*	22.7 ± 0.64	25.71 ± 0.57*	24.27 ± 0.78	23.7 ± 0.66
Non-HDL-C*f*	39.17 ± 2.03	52.28 ± 2.41**	42.9 ± 2.43#	57.64 ± 2.37***

f mg/dl. Values are mean ± SEM;

* *P* < 0.05,

** *P* < 0.01,

*** *P* < 0.001 as compared with the N + GNO diet;

# *P* < 0.05,

## *P* < 0.01 as compared to the D + GNO diet (by one-way ANOVA followed by Dunnett’s multiple comparison test); N + GNO = normal rats fed with groundnut oil; D + GNO = diabetic rats fed with groundnut oil; D + CO = diabetic rats fed with coconut oil; D + PO = diabetic rats fed with palm oil.

### Effect on the liver antioxidant enzymes and LPO

D-induced rats showed a significant drop in SOD while they had elevated LPO. However, D control rats did not show significant changes in catalase as compared to the N + GNO group. Among the two SFA-rich dietary fats studied, the D + PO group showed a significant decrease in SOD (*P* < 0.001) and an increase in LPO (*P* < 0.01) whereas the CO-treated group produced no changes in these two parameters. Furthermore, PO administration did not significantly alter the D-associated changes. The CO seemed to have apparently reversed the D-induced changes in LPO and antioxidant status [Table 2].

**Table 2:** Effect of saturated fatty acid-rich dietary vegetable oils on antioxidant enzymes in normal and diabetic rats (n = 6)

*Parameters*	*N* + *GNO*	*D* + *GNO*	*D* + *CO*	*D* + *PO*
SOD∆	32.39 ± 0.6	27.84 ± 0.8**	30.39 ± 0.6	26.04 ± 0.9***
Catalase∆	175.7 ± 4.8	163.3 ± 4.9	172.2 ± 3.5	160.8 ± 4.9
Lipid peroxidation∇	1.32 ± 0.04	1.56 ± 0.07*	1.50 ± 0.03	1.63 ± 0.06**

∆ U/mg of protein,

∇ nM MDA/mg of protein. Values are mean ± SEM;

* *P* < 0.05,

** *P* < 0.01,

*** *P* < 0.001 as compared to the N + GNO diet (by one-way ANOVA followed by Dunnett’s multiple comparison test); SOD = superoxide dismutase; N + GNO = normal rats fed with groundnut oil; D + GNO = diabetic rats fed with groundnut oil; D + CO = diabetic rats fed with coconut oil; D + PO = diabetic rats fed with palm oil.

### Effect on oral glucose tolerance

**Figure 2:**
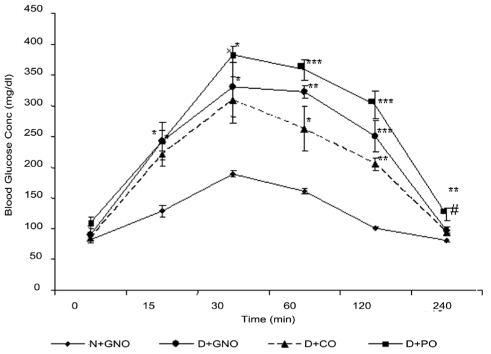
Effect of saturated fatty acid-rich dietary vegetable oils on blood glucose levels during oral glucose tolerance test in diabetic rats (n = 6). *P < 0.05, **P < 0.01, ***P < 0.001 as compared with the N + GNO diet. #*P* < 0.05 as compared to the D + GNO diet (by one-way ANOVA followed by Dunnett’s multiple comparison test); N + GNO = normal rats fed with groundnut oil; D + GNO = diabetic rats fed with groundnut oil; D + CO = diabetic rats fed with coconut oil; D + PO = diabetic rats fed with palm oil

All D groups had high glucose levels following OGTT when compared to N animals. Among the three groups, the CO-administered group showed minimal rise in the blood glucose level. The PO-administered group showed consistently high sugar levels at all the time intervals of sugar monitoring [Figure 2].

## Discussion

The present study shows that CO has a beneficial effect against the DM-associated dyslipidemia, oxidative stress and glucose intolerance, thereby exhibiting a trend toward anti-atherosclerotic profile. On the contrary, PO has a trend toward pro-atherosclerotic profile. It has been reported in the literature that CO possesses many of the health benefits, viz. hypocholesterolemic, hypolipemic, antiplaque, antioxidant[3] and antidiabetic[12,13] properties. One of the reasons could be the difference in chain lengths that are metabolized differently. The major FA content of CO is medium-chain lauric acid (48%).[3,9] Lauric acid (C 12:0) gets converted into monolaurins – the best fat similar to mother’s milk.[3,9] The short- and medium-chain SFAs are easily digested, absorbed and utilized by the body and contribute less fat deposition when compared to long-chain fatty acids (LCFAs).[14] LCFAs, however, are transported via chylomicrons into the lymphatic system, allowing for extensive uptake into the adipose tissue, as evident in our study, by the hypolipedemic activity of CO in D rats.

The present study has also observed aggravated D-associated dyslipidemia [Table 1] and oxidative stress [Table 2] in the PO-fed group. PO, an SFA dietary oil, contains 40% of palmitic acid (C16:0) and only 0.2% lauric acid.[2,15] Palmitic acid is suspected to possess a hypercholesterolemic effect.[16] It increases the cholesterol ester transport protein activity, which is responsible for the transfer of cholesterol from HDL to LDL. This, in turn, is responsible for the decrease in the HDL-C concentration combined with an increase in LDL-C.[17] Moreover, palmitic acid is metabolized to palmitoleic acid, which, in turn, reduces HDL-C.[18] We also observed an increase in the non-HDL-C levels and AI in the PO-treated D group, which further confirms the harmful effects of palmitic acid-rich PO on health. The increase in the non-HDL-C level and oxidative stress in the PO-fed group may result in the formation of more oxidized LDL-C, which is known to accelerate the atherosclerosis process. The beneficial effects of CO and the deleterious effects of PO were also observed in our earlier study involving N and stressed rats.[2] Hence, it can be suggested that the deleterious effects observed by the SFA-rich oil is dependent on the type of FAs present.

Results of OGTT have shown better glucose tolerance with CO whereas PO has impaired glucose tolerance. The observed difference in insulin sensitivity between two SFA-containing oils studied may be related to the TG level. There are reports on the association of elevated serum TGs with insulin resistance. As per Storlien *et al*., one of the mechanisms for the fat-induced insulin resistance could be attributed to an increased accumulation of TG in the skeletal muscle.[19] Besides, several other studies involving animals and humans have shown that the FA composition of muscle membrane phospholipid is closely associated with insulin action, altering fluidity and receptor activity.[20,21] The difference in insulin sensitivity between two SFA-containing oils studied may also be due to the change in the composition of FA in the membrane phospholipids. It has been reported earlier that CO, due to the increased content of lauric acid, improves insulin sensitivity and reduces the incidence of DM.[13,14] But, peripheral insulin sensitivity was significantly and negatively correlated with the proportion of palmitic and palmitoleic acids.[21] Amelsvoort *et al*. have also reported that PO feeding resulted in a lower rate of insulin-stimulated glucose uptake and insulin binding to the cells (lower number of low-affinity binding sites) than feeding sunflower oil.[22] Therefore, based on our results, we can state that the insulin insensitivity due to the higher intake of saturated fat may be relevant to palmitic acid-rich oil but may not be due to lauric acid-rich CO.

The present study claims the protective role of lauric acid-rich CO against oxidative stress, dyslipidemia and glucose intolerance. PO due to low content of lauric acid and high content of palmitic acid was found to manifest deleterious effects of SFA. Hence, consumption of high amount of lauric acid- and less amount of palmitic acid-containing oils is likely to provide marked improvement in many aspects of the metabolic syndrome via a range of direct and indirect effects on glucose/lipid metabolism in the body against DM and DM-associated complications. It can be concluded that it is not just SFA but also the type of FA (lauric acid) present in the dietary oil used that is important.
